# Efficiency in the Endoscopy Unit: Can We ‘Turn Around’ Room Turnover? An Observational Quality Improvement Study

**DOI:** 10.1093/jcag/gwac005

**Published:** 2022-03-10

**Authors:** Carolyn Michelle Tan, Michael Bernstein, Janet Raboud, Benedetta Mannino, Jill Tinmouth

**Affiliations:** Department of Medicine, University of Toronto, Toronto, Ontario, Canada; Department of Medicine, Sunnybrook Health Sciences Centre, Toronto, Ontario, Canada; Department of Medicine, University of Toronto, Toronto, Ontario, Canada; Department of Medicine, Sunnybrook Health Sciences Centre, Toronto, Ontario, Canada; Toronto General Hospital Research Institute, University Health Network, Toronto, Ontario, Canada; Dalla Lana School of Public Health, University of Toronto, Toronto, Ontario, Canada; Department of Medicine, Sunnybrook Health Sciences Centre, Toronto, Ontario, Canada; Department of Medicine, University of Toronto, Toronto, Ontario, Canada; Department of Medicine, Sunnybrook Health Sciences Centre, Toronto, Ontario, Canada; Ontario Health (Cancer Care Ontario), Toronto, Ontario, Canada; Institute for Health Policy, Management and Evaluation, University of Toronto, Toronto, Ontario, Canada

**Keywords:** Endoscopy, Efficiency, Turnover time, Non-procedure time, Quality improvement

## Abstract

**Background:**

Endoscopy units are being challenged to provide timely and quality care, despite limited resources and an ever-growing patient population. Decreasing procedure time is unlikely to create sufficient time savings and may compromise quality. Non-procedural factors, such as room turnover, are important contributors to efficiency and represent an ideal target for quality improvement efforts.

**Aims:**

The objective of this quality improvement study was to identify practices that will improve endoscopy unit efficiency at our centre. The specific aims were to (a) understand practices at local hospitals that contribute to room turnover efficiency and (b) examine the magnitude and sources of variation in room turnover efficiency across endoscopists and nurses at our centre.

**Methods:**

Interviews were conducted with team leads at five local hospitals. Routinely collected data from our centre were analyzed to understand the magnitude and variation in efficiency by provider and reasons for delays. Non-procedure time defined as ‘patient 1 scope out’ to ‘patient 2 scope in’ was our primary measure of efficiency.

**Results:**

Over the 12-month period, 750 outpatient procedures met inclusion criteria. Median non-procedure time was 19 min (interquartile range: 16–22 min). The variation attributable to endoscopists was 14.7% compared to 80.4% for unmeasured factors.

**Conclusions:**

The variation that remains unexplained by our model suggests that unmeasured factors play a substantial role in endoscopy unit efficiency and that our current endoscopy records are not capturing important contributors to efficiency. The next phase will involve focus groups and direct observation with the goal of identifying these unmeasured factors.

## INTRODUCTION

Over the last two decades, quality has become a key focus for gastroenterology, with many efforts to define, measure and improve quality in endoscopic procedures (1). Although quality is important, there is also a need to focus on efficiency. Approximately 50% of patients wait double the recommended maximum wait times for a diagnostic colonoscopy after having a positive colorectal cancer screening test (2,3). Prolonged wait times are associated with a greater risk of colorectal cancer and more advanced disease at the time of diagnosis (4).

Endoscopy units are being challenged to provide timely and quality care, despite limited resources and an ever-growing patient population (5). The strain on the system has been exacerbated by a substantial backlog of endoscopy procedures due to the COVID-19 pandemic (6). Audits of endoscopy units have revealed significantly underutilized resources, making the measurement and optimization of efficiency an ideal target for quality improvement (QI) efforts (7).

Earlier studies found that decreasing procedure time is unlikely to save sufficient time to allow additional cases to be scheduled (8). Procedure time is also not an ideal focus because shorter procedure times may compromise quality (9–11). Non-procedure-related factors, on the other hand, have been identified as important contributors to endoscopy unit efficiency (9,12,13) and therefore represent key targets for improvements in efficiency (14).

Improving endoscopy unit efficiency is a multidisciplinary endeavour (7,9,14,15). However, to the best of our knowledge, no studies have examined the contributions of individual endoscopists and nurses to non-procedural determinants of efficiency such as room turnover time.

The overall objective of this QI study was to identify potentially modifiable practices that will improve endoscopy unit efficiency for routine outpatient gastrointestinal (GI) procedures in a single center in Toronto, Canada. The specific aims of this study were to (a) understand local practices that contribute to room turnover efficiency and (b) examine the magnitude and sources of variation in room turnover efficiency across endoscopists, nurses and endoscopist-nurse pairs.

## METHODS

The Strengthening the Reporting of Observational Studies in Epidemiology (STROBE) guidelines were followed during the reporting of this study. As per the Sunnybrook Health Sciences Centre (SHSC) Ethics Review Self-Assessment Tool, research ethics board approval was not required for this QI study.

### Aim 1: Local Hospital Practice

#### Overview

To gain a better understanding of local practices and any initiatives undertaken to measure and improve room turnover efficiency, telephone interviews were conducted with endoscopy unit team leads at five nearby hospitals. Understanding local context and best practices is important as it may help identify process changes that can be adapted to SHSC and determine if our organization’s benchmark for efficiency is similar to that of other sites.

#### Participants

Five endoscopy units in Toronto, Canada were invited to participate, and all completed the survey; there were three academic and two community hospitals. Nursing team leads from each unit were recruited via email.

#### Data Collection

We conducted 30- to 45-min telephone interviews with the nursing team leads at four units using our Toronto Endoscopy Survey (Supplementary Appendix), which included questions about endoscopy unit staffing, room turnover time, delays and initiatives undertaken to improve efficiency. Questions were read exactly as written in the survey. The team lead at the fifth unit completed a paper copy of the survey instead.

### Aim 2: Variation in Endoscopy Efficiency

#### Overview

For the second aim, we used data routinely collected during endoscopy over a period of 1 year to understand the magnitude and variation in the time between procedures by provider type and their interaction as well as reasons for delays. Although five hospitals were surveyed to provide local context, this quantitative analysis was only conducted at our center, SHSC.

#### Setting

SHSC is a tertiary referral hospital with 690 inpatient acute beds. The endoscopy unit consists of two standard procedure rooms and a recovery room; there is a third room which was used almost exclusively for advanced procedures at the time of the study. Two endoscopists work simultaneously such that each only has access to a single room at a time which must be turned over before the next case.

The two standard rooms are staffed with one endoscopy nurse each while the 9-bed recovery room is staffed by two nurses. There is a single float nurse who moves between the two rooms and the recovery room. Seven gastroenterologists and four general surgeons routinely use the unit. Gastroenterology trainees are frequently involved in procedures, but their involvement is not recorded in the computer system so their influence on efficiency cannot be assessed.

Each individual physician is allocated ‘booking time’ for their procedures, based on the mean time required for their most recent 10 cases (by procedure type), excluding the shortest and longest cases. Since 2015, endoscopy nurses have been recording data using the computer software, Picis Clinical Solutions (Picis). For each case, information recorded includes the endoscopist and nurse(s) involved, procedure start and end times, the time at which patients are brought into and out of the endoscopy room, and reasons for delay. Institutional targets are 5 min for the time from ‘patient 1 out’ to ‘patient 2 in’ and 15 min for the time from ‘patient 1 scope out’ to ‘patient 2 scope in’.

#### Participants

The cohort comprised all persons having routine outpatient endoscopic procedures (e.g., colonoscopy, oesophagogastroscopy, flexible sigmoidoscopy) from April 1, 2018 to March 31, 2019. If an individual had more than one procedure during the study period, only the first was included. Respirology, inpatient, advanced endoscopic (e.g., endoscopic retrograde cholangiopancreatography and endoscopic ultrasound), planned therapeutic and cases involving anesthesia were excluded. Planned therapeutic cases were defined as those where a therapeutic intervention such as argon plasma coagulation, dilation or insertion of a percutaneous endoscopic gastrostomy tube was planned in advance.

**Figure 1.**
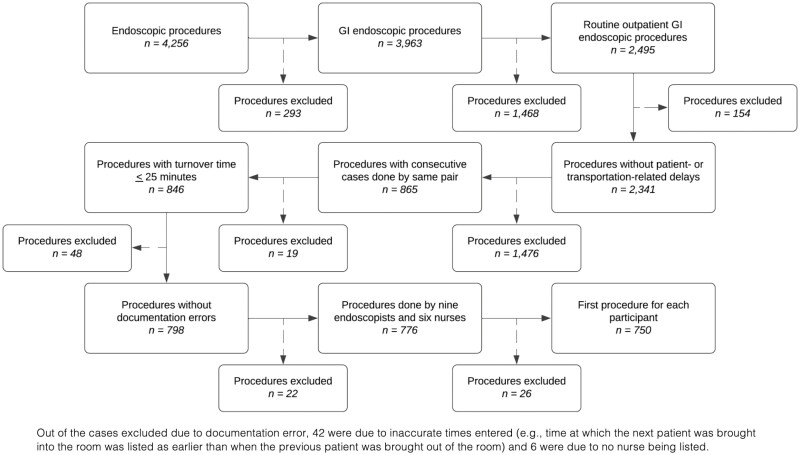
Flow diagram showing procedure selection.

Based on experience, it was thought that human factors such as the interaction between endoscopists and nurses would impact efficiency. Thus, we sought to evaluate efficiency by endoscopist-nurse pair, excluding consecutive cases that were not performed by the same pair (Figure 1). Cases by endoscopists or nurses who completed fewer than 20 cases during the study period were also excluded. The inclusion of staff who work infrequently in the unit may lead to results that are not generalizable to the majority of endoscopists and nurses in our unit. Given that our aim was to identify modifiable practices for our endoscopy unit personnel, procedures affected by patient- and transportation-related delays were excluded. Lastly, any case with a turnover time > 25 min was excluded since this was more likely due to a scheduled break.

#### Definitions

##### Outcomes

Given the focus of this study on non-procedural factors, we defined endoscopy unit efficiency as the avoidance of time waste between procedures. We measured this in two ways: ‘turnover time’ (TT), defined as ‘patient 1 out’ to ‘patient 2 in’, and ‘non-procedure time’ (NPT), defined as ‘patient 1 scope out’ to ‘patient 2 scope in’. NPT encompasses all the important processes that occur outside the procedure and is arguably less susceptible to differences in practice across endoscopy unit personnel (i.e., some complete paperwork once the patient has left the room, others complete it while the patient is in the room).

##### Factors Considered

The primary factor of interest was the contribution of endoscopists and nurses to NPT and TT, while accounting for procedure type, procedure complexity, time of day (mornings vs. afternoon cases) and incomplete consents. Number of pathology containers was used as a surrogate for procedure complexity. Morning cases were defined as those where the patient was brought into the endoscopy room before 1200h.

##### Delays

The nurse is responsible for recording the reasons for delay in Picis; these were divided into five major categories (Table 1). Incomplete consents were separated out from other endoscopist-related delays because they represented a significant proportion of all delays.

**Table 1.** Definitions of time intervals and delays

Delay	Definition
Endoscopist-related	Endoscopist unavailable to start the procedure at the scheduled time (e.g., performing same-day consultation or away from the endoscopy room)
Nurse-related	Nurse unavailable to start the procedure at the scheduled time
Consent incomplete	Procedure started after scheduled time due to endoscopist obtaining consent
Equipment-related	Equipment not available
System-related	Incorrect bookings, room unavailability due to emergency case, prolonged housekeeping clean-up

##### Statistical Methods

Characteristics of procedures and participants were reported using frequencies and percentages for categorical variables and medians and interquartile ranges (IQRs) for continuous variables. A heat map was used to illustrate the variation in median NPT and TT across endoscopist-nurse pairs.

Generalized linear mixed effects models were used to identify associations of NPT and TT with the following independent variables: endoscopist, nurse, procedure type, number of pathology containers (categorized as 0, 1–2, 3+), time of day (morning vs. afternoon) and incomplete consent. Procedures were grouped as follows: colonoscopy, oesophagogastroduodenoscopy (OGD), OGD-colonoscopy and OGD-sigmoidoscopy, and other (flexible sigmoidoscopy, proctoscopy, pouchoscopy and ileoscopy).

Random effects were used to model the effects of endoscopist and nurse. Fixed effects were used for the remaining independent variables. Age, sex and ASA were thought to unlikely be important a priori but were included in a sensitivity analysis. Models were fit using the *lmer* procedure in R (16). QQ plots were used to examine the distribution of residuals from the models. Since the distributions differed significantly from normal at the tails, square root transformations were considered for NPT and TT. We present results for the untransformed variables.

## RESULTS

### Aim 1: Local Hospital Practice

Findings from the survey of five local hospitals compared to SHSC are shown in Table 2. Most notably, three allocated 5 min for turnover and two allocated 10 min for turnover. All centers reported tracking TT and stated that observed TT was the same as the allocated time. All five centers make use of support staff to help with room turnover and four reported undertaking initiatives to decrease TT.

**Table 2.** Anonymized key survey findings from five Toronto hospitals compared to SHSC

Type of hospital	Hospital 1	Hospital 2	Hospital 3	Hospital 4	Hospital 5	SHSC
	Community	Academic	Community	Academic	Academic	Academic
Endoscopist-to-room ratio	1:1	1:1	1:1	1:1	1:1	1:1
Measure non-procedure time?∗	No	No	No	No	No	Yes
Measure turnover time?†	Yes	Yes	Yes	Yes	Yes	Yes
Allocated turnover time	10 minutes	5 minutes	5 minutes	5 minutes	10 minutes	5 minutes
Observed turnover times similar to allocated times?‡	Yes	Yes	Yes	Yes	Yes	Yes
Estimated average # of procedures per room per day‡	15	11-12	15	9-10	13	10
Delays documented?	Only for first case of the day	Yes	Yes	Yes	Yes	Yes
Delays reviewed?	No	Yes (monthly)	Yes (quarterly or as necessary)	Yes (monthly)	Yes (monthly)	No
Support staff§ to help with room turnover and patient transport	3 team attendants	1 housekeeper	1 team attendant	3 team attendants	3 housekeepers	1 porter
Initiatives taken to improve efficiency	Yes (team attendant added)	No	Yes (involvement of flow team, consent process standardized, team attendant added)	Yes (team attendant and float nurse added)	Yes (team lead has 1-on-1 meetings with nurses and housekeepers every 2-3 months)	No
Performance data shared with staff?	No	Yes (aggregate only)	Yes (aggregate and individual)	No	Yes (aggregate and individual)	No

Defined as ‘patient 1 scope out’ to ‘patient 2 scope in’.

Defined as ‘patient 1 out’ to ‘patient 2 in’.

Estimate provided by nursing team lead.

Roles of support staff varied across hospitals. In general, housekeepers helped with cleaning only, porters helped with transport only, and team attendants helped with both.

### Aim 2: Variation in Endoscopy Efficiency

Over the 12-month period, there were 4256 endoscopic procedures performed in the SHSC endoscopy unit, of which 3963 were GI endoscopic procedures. Routine outpatient GI endoscopic procedures comprised 2495 and of these, 1476 were excluded as they were not consecutive cases performed by the same endoscopist-nurse pair and 269 for other reasons. We included 750 consecutive cases, performed by nine endoscopists (seven gastroenterologists and two general surgeons) and six nurses (Figure 1).

The characteristics of endoscopists and nurses including years of experience, gender and number of procedures performed are included in Table 3. Number of cases performed by endoscopist-nurse pairs ranged from 0 to 47. Median patient age was 65 years (IQR 22 years) with 53% female patients and a median ASA class of II (range I–IV). Characteristics of the included cases by procedure type are shown in Table 4.

**Table 3.** Characteristics of included endoscopists and nurses

Operator	Experience, median, (IQR), years	Female, no. (%)	Procedures∗, median, (IQR), no.
Endoscopists (9)	17 (13-21)	4 (44%)	74 (48–116)
Nurses (6)	11 (9-17)	5 (83%)	128 (94–155)

From April 1, 2018 to March 31, 2019.

**Table 4.** Characteristics of included procedures

Type of procedure	Patients, no. (%)	Median pathology containers, no. (IQR)	Median NPT (IQR), min	Median TT (IQR), min
Colonoscopy	345 (46%)	1 (0–2)	18 (15–22)	6 (4–9)
OGD	230 (31%)	1 (0–2)	20 (17–23)	7 (5–10)
OGD-sigmoidoscopy/colonoscopy	80 (11%)	2 (1–3)	20 (18–24)	7 (5–10)
Other∗	95 (13%)	0 (0–1)	16 (13–20.5)	6 (3–8.5)
All	750 (100%)	1 (0–2)	19 (16–22)	6 (4–9)

Flexible sigmoidoscopy, ileoscopy, pouchoscopy, and proctoscopy. OGD, oesophagogastroduodenoscopy.

### Non-Procedure Time

**Figure 2.**
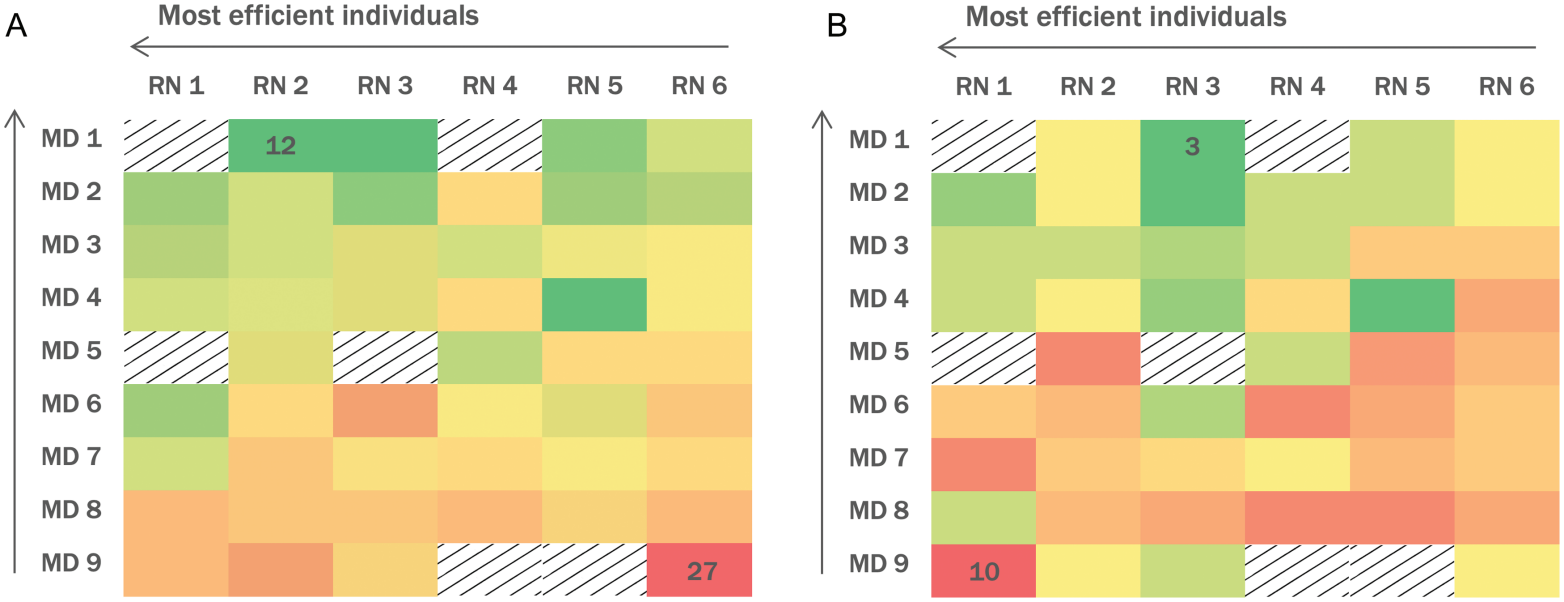
Median NPT (left) and TT (right) in minutes for each endoscopist-nurse pair. The fastest times are represented in light grey, with times progressively increasing as the colors change from darker grey to black. Pairs that completed < 3 cases together are represented by a box with lines. MD and RN number assignments are the same in both heat maps.

The median NPT for the unit was 19 min (IQR 16–22 min, min–max 6–55 min) (Supplementary Appendix, Table 1). Individual median NPTs for our nine endoscopists ranged from 13.5 to 23 min, whereas median NPTs for our six nurses ranged from 17 to 19 min. Median NPT for each endoscopist-nurse pair ranged from 12 to 27 min (Figure 2). There were 563 cases (75%) with NPTs over the target of 15 min and 272 cases (36%) with NPTs greater than 5 min over the target.

The variation in NPT attributable to endoscopists was 14.7%, 1.4% for nurses and 3.5% for the combined fixed effects (Supplementary Appendix, Table 2). Unmeasured variation accounted for 80.4% of NPT variation. Compared to colonoscopy, NPT was longer for OGD-sigmoidoscopy and OGD-colonoscopy by 3.05 min (*P* < 0.0001) and for OGD by 1.27 min (*P* = 0.01; Table 5). The number of pathology containers, time of day and incomplete consents were not significantly associated with NPT. Sensitivity analysis confirmed that as suspected a priori, age, sex and ASA did not materially change the results.

**Table 5.** Fixed effects estimates from multivariable generalized linear mixed model for non-procedure time∗

	Coefficient Estimate † (95% CI)	*P*-value
Consent Incomplete	1.34 (−0.21, 2.87)	0.08
(Reference = consent complete)		
Pathology containers (Reference = none)		
1-2 pathology containers	−0.023 (−0.90, 0.86)	0.96
3 or more pathology containers	0.33 (−0.84, 1.51)	0.58
Procedure (Reference = colonoscopy)		
OGD	1.27 (0.32, 2.24)	0.01
OGD-sigmoidoscopy/colonoscopy	3.05 (1.71, 4.41)	<0.0001
Other ‡	–0.55 (–1.83,0.72)	0.40
Time of day (Reference = morning)		
Afternoon	0.54 (–0.45, 1.50)	0.28

Random effects were used for physician and nurse.

The coefficient estimate represents the number of minutes in NPT associated with each covariate.

Flexible sigmoidoscopy, ileoscopy, pouchoscopy, and proctoscopy.

OGD, oesophagogastroduodenoscopy.

### Turnover Time

The overall median TT for the unit was 6 min (IQR 4–9 min, min–max 0–25 min; Supplementary Appendix, Table 3). Individual median TTs ranged from 5 to 8 min for each endoscopist and 5 to 7 min for each nurse. Median TT for each endoscopist-nurse pair ranged from 3 to 10 min (Figure 2). There were 450 cases (60%) with TTs over the target.

The variation in TT attributable to endoscopists was 3.9%, 1.4% for nurses and 1.5% for all fixed effects combined (Supplementary Appendix, Table 4). Unmeasured factors accounted for 93.2% of TT variation. TT was longer in the afternoon by 0.84 min (*P* = 0.022) and by 1.06 min for OGD sigmoidoscopy and OGD colonoscopy relative to colonoscopy (*P* = 0.045; Table 6). The number of pathology containers and incomplete consents did not significantly affect TT. Sensitivity analysis confirmed that as suspected a priori, age, sex and ASA did not materially change the results.

**Table 6.** Fixed effects estimates from multivariable generalized linear mixed model for turnover time∗

	Coefficient estimate † (95% CI)	*P*-value
Consent Incomplete	0.29 (−0.86, 1.44)	0.61
(Reference = consent complete)		
Pathology containers (Reference = none)		
1-2 pathology containers	−0.24 (−0.91, 0.43)	0.48
3 or more pathology containers	−0.46 (−1.35, 0.44)	0.31
Procedure (Reference = colonoscopy)		
OGD	0.24 (−0.48, 0.99)	0.51
OGD-sigmoidoscopy/colonoscopy	1.06 (0.03, 2.10)	0.045
Other ‡	−0.28 (−1.25,0.70)	0.58
Time of day (Reference = morning)		
Afternoon	0.84 (0.09, 1.55)	0.02

Random effects were used for physician and nurse.

The coefficient estimate represents the number of minutes in TT associated with each covariate.

Flexible sigmoidoscopy, ileoscopy, pouchoscopy, and proctoscopy.

OGD, oesophagogastroduodenoscopy.

### Excluded Cases

Many consecutive cases were excluded because they were not performed by the same endoscopist-nurse pair (Figure 1). The distribution of procedure types was similar for included and excluded cases (Supplementary Appendix, Table 5). Median NPT and TT were 1 min longer compared to consecutive cases performed by the same pairs (median NPT: 20 min [IQR 16–26 min] and median TT: 7 min [IQR 4–11]).

### Delays

**Figure 3.**
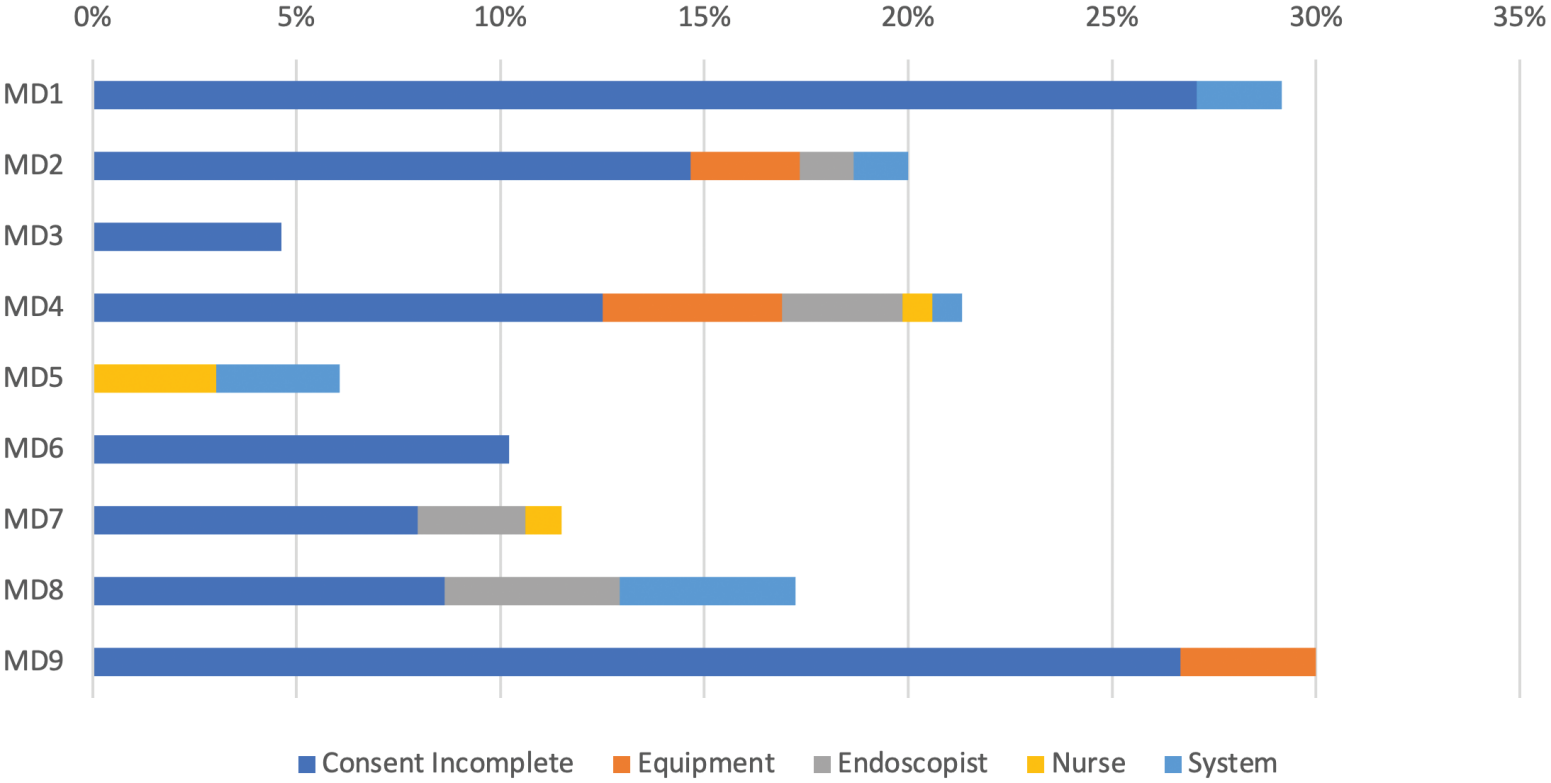
Percentage of cases affected by delays sorted by an endoscopist.

Out of the 750 included consecutive case-pairs, 114 (15.2%) were affected by delays. The most common delay was incomplete consent which affected 80 cases (10.7%). The least common was nurse-related delay which affected 3 cases (0.4%). Of the delayed cases, 81.6% were endoscopist-related. The distribution of the different types of delays by endoscopist is illustrated in Figure 3.

## DISCUSSION

In our center, we found substantial variation in NPT across endoscopists, with the most efficient having a median NPT nearly 10 min faster than the least efficient. Although endoscopist and procedure type were significantly associated with NPT, the greatest variation was in residual error, suggesting that unmeasured factors play the largest role in non-procedural endoscopy unit efficiency. The important role of unmeasured factors is further substantiated by the lack of correlation between endoscopist efficiency and recorded delays. These factors may be better identified and evaluated in the next phase of our study, which will include direct observation. Furthermore, our findings suggest that NPT, as defined in our study, should be used instead of TT to determine non-procedural efficiency.

Three-quarters of the cases in our unit had NPTs over the target of 15 min. Achieving the target of 15 min may create sufficient time savings for one additional procedure per room each day. Over the course of a year, this would translate to approximately 250 additional procedures per room, or 500 for the entire unit.

In our study, endoscopists accounted for the largest proportion of measurable error. Our work builds on prior work by Yong et al. which had identified endoscopist-related delays as a major source of inefficiency. Zamir and Rex similarly found that an endoscopist’s mean NPT was the strongest predictor of endoscopist efficiency. Although earlier studies highlighted the importance of non-procedural determinants of endoscopist and endoscopy unit efficiency, this was the first study to examine the magnitude and sources of variation in NPT across endoscopists, nurses and pairs.

Endoscopist-related delays accounted for over 80% of all delays. Incomplete consents were the most common (Figure 3), yet they were not associated with NPT, possibly because of the variation in time spent obtaining informed consent. Nonetheless, the high proportion of cases delayed by incomplete consents suggests that this may be an important area of focus. Standardizing the consent process was also a priority for Hospital 3, the only center that hired a flow team to identify areas for improvement.

Endoscopy unit efficiency is acknowledged as an important issue as all endoscopy units surveyed track TT and four of five units have undertaken initiatives to improve efficiency. In the literature, TT is one of the most commonly reported measures of efficiency, yet it is inconsistently defined. Some studies define it as ‘patient 1 out’ to ‘patient 2 in’ (5,7,12), whereas others define it as ‘patient 1 scope out’ to ‘patient 2 scope in’ (9,13). The former may be inadequate as it only captures a portion of the time and tasks performed between procedures; furthermore, our findings suggest that it is not an accurate surrogate for NPT. As demonstrated in Figure 2, the pairs with the fastest TTs did not necessarily have the fastest NPTs, and likewise for the slowest. Our study is the first to make this distinction between TT and NPT and highlights potential pitfalls of using TT as a marker of efficiency.

Although barriers to efficiency will vary across endoscopy units, we believe that the benefits of using NPT over TT as a measure of efficiency, as well as the importance of considering unmeasured factors, are two important findings that are generalizable to other endoscopy units.

### Limitations

Limitations include those inherent to studies using routinely collected data. First, delay codes are recorded by nurses which may affect how frequently the “nurse not available” delay code is used. Second, cases with TT > 25 min were excluded since they more likely represent a scheduled break, but the sensitivity and specificity of this cut-off are unclear. Third, the time of ‘patient out’ reflects an estimate by the nurse since this must be entered before the endoscopy record can be printed and the patient can be brought to recovery. Using NPT instead of TT overcomes this limitation as it would not be affected by time of ‘patient out’. Fourth, involvement of trainees is not recorded in Picis so their influence on NPT and TT could not be assessed. However, in their prospective study, Yong et al. found that trainee involvement in our unit was associated with increased procedure duration but not increased TT, suggesting that trainees would have had little to no effect on NPT and TT (13).

Usually there is only one endoscopy nurse per room. Occasionally, a second nurse will help with the turnover process, but their name is often not added to the endoscopy record. It is uncertain whether this practice is applied equally across endoscopists, or between nurses; if not, it could be a source of unmeasured confounding. It is also possible that endoscopists with shorter booking times receive more help so that the room stays on schedule, resulting in a self-fulfilling prophecy. Similarly, if an endoscopist with longer booking times finishes early, their next patient may not be ready, reducing the need to turn over the room efficiently. The above limitations related to using routinely collected data will be addressed in the next phase of our study which involves direct observation.

## CONCLUSIONS

There is substantial variation in NPT across endoscopists, yet the variation that remains unexplained by our model suggests that there are unmeasured factors affecting endoscopy unit efficiency. In other words, there appear to be important contributors to efficiency that are not being captured or monitored by our current endoscopy records. The next phase of this study will involve focus groups and direct observation of cases with the goal of identifying these unmeasured factors. Particular attention will be given to the most and least efficient endoscopists and endoscopist-nurse pairs. It is anticipated that these findings will enable us to discover efficient practices that can be implemented using QI methods, with the goal of improving NPT, procedure volumes, and ultimately patient wait times and outcomes in our unit.

## Supplementary Material

gwac005_suppl_Supplementary_Appendix
