# Supplementary material for: Efficiency in the Endoscopy Unit: Can We ‘Turn Around’ Room Turnover? An Observational Quality Improvement Study
Source: J Can Assoc Gastroenterol. 2022 Mar 10;5(4):e57–64. doi: 10.1093/jcag/gwac005 (PMC9340627; doi:10.1093/jcag/gwac005)
Supplement: gwac005_suppl_Supplementary_Appendix [file gwac005_suppl_supplementary_appendix.docx]

**APPENDIX**

Table 1. Summaries of NPT by covariate

|  |  | N | Min | Q1 | Mean | Median | Q3 | Max |
| --- | --- | --- | --- | --- | --- | --- | --- | --- |
| MD | | | | | | | | |
|  | MD1 | 48 | 8 | 11.00 | 15.10 | 13.5 | 18.25 | 29 |
|  | MD2 | 74 | 6 | 14.00 | 17.70 | 16.5 | 21.00 | 40 |
|  | MD3 | 151 | 10 | 15.00 | 18.09 | 17.0 | 21.00 | 36 |
|  | MD4 | 136 | 11 | 15.00 | 19.18 | 18.0 | 21.25 | 48 |
|  | MD5 | 33 | 11 | 17.00 | 18.94 | 18.0 | 21.00 | 29 |
|  | MD6 | 49 | 10 | 16.00 | 20.65 | 19.0 | 25.00 | 55 |
|  | MD7 | 113 | 9 | 17.00 | 20.15 | 20.0 | 23.00 | 40 |
|  | MD8 | 116 | 12 | 19.00 | 22.25 | 21.0 | 25.00 | 42 |
|  | MD9 | 30 | 12 | 19.25 | 23.77 | 23.0 | 26.75 | 43 |
| Procedure type | | | | | | | | |
|  | Colonoscopy | 345 | 6 | 15 | 18.68 | 18 | 22.0 | 41 |
|  | OGD | 230 | 10 | 17 | 20.49 | 20 | 23.0 | 43 |
|  | OGD-sigmoidoscopy/  colonoscopy | 80 | 11 | 18 | 21.93 | 20 | 24.0 | 55 |
|  | Other* | 95 | 8 | 13 | 17.57 | 16 | 20.5 | 48 |
| Time of day | | | | | | | | |
|  | AM | 410 | 6 | 16 | 19.80 | 19 | 22 | 48 |
|  | PM | 340 | 8 | 15 | 19.00x | 18 | 22 | 55 |
|  |  |  |  |  |  |  |  |  |
| All | | 750 | 6 | 16 | 19.44 | 19 | 22 | 55 |

*Flexible sigmoidoscopy, ileoscopy, pouchoscopy, and proctoscopy

OGD = oesophagogastroduodenoscopy.

Table 2. Sources of variation in generalized linear mixed model of NPT

| Source of Variation | Variance | Standard Deviation and 95% CI | Percentage of Variation |
| --- | --- | --- | --- |
| Random Effects  MD  Nurse | 5.27  0.50 | 2.29 (1.30,3.89)  0.70 (0, 1.77) | 14.7%  1.4% |
| Fixed Effects | 1.28 |  | 3.5% |
| Residual | 28.84 | 5.37 (5.08, 5.63) | 80.4% |

**Marginal R^2^ = fixed var/(fixed + random + residual)**

**Conditional R^2^ = (fixed + random)/(fixed + random + residual)**

**Conditional ICC = random/(random + residual)**

Table 3. Summaries of TT by covariate

|  |  | N | Min | Q1 | Median | Mean | Q3 | Max |
| --- | --- | --- | --- | --- | --- | --- | --- | --- |
| MD | | | | | | | | |
|  | MD1 | 48 | 0 | 3.0 | 6.00 | 5.0 | 8.0 | 22 |
|  | MD2 | 74 | 0 | 4.0 | 5.97 | 5.0 | 8.0 | 18 |
|  | MD3 | 151 | 0 | 4.0 | 6.41 | 6.0 | 8.5 | 20 |
|  | MD4 | 136 | 0 | 4.0 | 7.16 | 6.5 | 9.0 | 22 |
|  | MD5 | 33 | 0 | 5.0 | 7.61 | 8.0 | 10.0 | 15 |
|  | MD6 | 49 | 0 | 5.0 | 8.33 | 7.0 | 11.0 | 19 |
|  | MD7 | 113 | 0 | 5.0 | 7.43 | 7.0 | 10.0 | 25 |
|  | MD8 | 116 | 0 | 5.0 | 8.25 | 8.0 | 10.0 | 22 |
|  | MD9 | 30 | 0 | 5.0 | 7.37 | 6.0 | 9.0 | 25 |
| Procedure type | | | | | | | | |
|  | Colonoscopy | 345 | 0 | 4 | 6.90 | 6 | 9.0 | 25 |
|  | OGD | 230 | 0 | 5 | 7.37 | 7 | 10.0 | 25 |
|  | OGD-sigmoidoscopy/  colonoscopy | 80 | 1 | 5 | 7.98 | 7 | 10.0 | 21 |
|  | Other* | 95 | 0 | 3 | 6.72 | 6 | 8.5 | 22 |
| Time of day | | | | | | | | |
|  | AM | 410 | 0 | 5 | 7.02 | 6 | 9 | 25 |
|  | PM | 340 | 0 | 4 | 7.26 | 7 | 10 | 25 |
| All | | 750 | 0 | 4 | 7.1 | 6 | 9 | 25 |

*Flexible sigmoidoscopy, ileoscopy, pouchoscopy, and proctoscopy

OGD = oesophagogastroduodenoscopy.

Table 4. Sources of variation in generalized linear mixed model of turnover time

| Source of Variation | Variance | Standard Deviation (95% CI) | Percentage of Variation |
| --- | --- | --- | --- |
| Random Effects  MD  Nurse | 0.70  0.26 | 0.84 (0.36,1.54)  0.51 (0, 1.19) | 3.9%  1.4% |
| Fixed Effects | 0.28 |  | 1.5% |
| Residual | 16.94 | 4.12 (3.90, 4.32) | 93.2% |

Table 5. Procedure types for included compared to excluded cases

| Type of procedure | Included cases, no. (%) | Excluded cases, no. (%) |
| --- | --- | --- |
| Colonoscopy | 345 (46%) | 297 (45%) |
| OGD | 230 (31%) | 176 (27%) |
| OGD-colonoscopy/sigmoidoscopy | 80 (11%) | 82 (13%) |
| Other* | 95 (13%) | 98 (15%) |

*Flexible sigmoidoscopy, ileoscopy, pouchoscopy, and proctoscopy

OGD = oesophagogastroduodenoscopy.

Toronto Endoscopy Survey

1. **Endoscopy unit**

- 1. What is the endoscopist-to-room ratio?
  - 1:1
  - 1:2
  - Other _____________________
  1. What are the unit operating hours and days?
     - 0800-1600h Monday to Friday
     - 0830-1630h Monday to Friday
     - Other ______________________
  2. Is there dedicated time each week for inpatients?
     - Yes (how much?)
     - No (please expand on how inpatient procedures are fit into the schedule)
  3. Excluding therapeutic and advanced procedures, what is the average number of outpatient procedures completed per room each day?

_____________________

1. **Staffing**
   1. How many endoscopists (i.e. gastroenterologists, general surgeons, nurse endoscopists) use the unit for outpatient procedures?

_____________________

- 1. How many full-time nurses and part-time nurses are there in total?

Full-time _______. Part-time _______

- 1. How many nurses are assigned to each endoscopy room for each type of routine procedure?

Gastroscopy _______ Colonoscopy _______ Flex sig _______

- 1. How many float nurses are there per day?

_____________________

- 1. Are certain nurses usually paired up with certain endoscopists?
     - Yes (please explain how this is decided)
     - No
  2. Aside from physicians and nurses, does the unit make use of any other support staff (e.g. environmental services, volunteers)?
     - Yes
     - No
     1. If YES, how many are there per shift and what are their responsibilities (e.g. gathering materials, cleaning rooms, transporting patients)?

1. **Training**
   1. Does the unit have standard operating procedures (SOPs) that describe each person’s responsibilities for room turnover, patient turnover, etc.?
      - Yes
      - No

3.1.1 If YES, do you have a copy of the SOPs that you can share with us?

SOPs available: Yes No

- 1. Is there a standardized training process for new endoscopy nurses?
     - Yes
     - No

3.2.1 If YES, please briefly describe the process

1. **Scheduling**
   1. Is the time allotted per procedure staff-dependent?
      - Yes
      - No
      1. If YES, how is this determined?
   2. How much time is allocated for each type of procedure? If yes to the previous question, please state the average amount of time allotted as well as the range

Gastroscopy: ______ minutes, (range if applicable: ______ minutes)

Colonoscopy: ______ minutes, (range if applicable: ______ minutes)

Flexible sigmoidoscopy: ______ minutes, (range if applicable: ______ minutes)

Gastroscopy plus flexible sigmoidoscopy: ______ minutes, (range if applicable: ______ minutes)

Gastroscopy plus colonoscopy: ______ minutes, (range if applicable: ______ minutes)

1. **Pre-procedure**

- 1. Are procedure start times being delayed by **physicians** completing paperwork (e.g. consents, history and physicals)?
     - Yes
     - No
     1. If YES, please expand (i.e. how often, type of paperwork, length of delay)
  2. Are procedure start times being delayed by **nurses** completing paperwork (e.g. intake form, pre-procedure notes, unfinished charting from previous procedure)?
     - Yes
     - No
     1. If YES, please expand (i.e. how often, type of paperwork, length of delay)

1. **Intra-procedure**
   1. Who documents information during the procedure and how is this done?

Documenter: _____________

Method:

- - - On paper
    - Using a computer program (please specify): _____________
    - Other: ______________
  1. What information, if any, is autopopulated? (e.g. vitals, procedure type, indication)
  2. When are pre-procedure vitals done? Check ALL that apply.
     - During intake
     - In the endoscopy room
     - Other: ______________
  3. Are delays documented?
     - Yes (please explain what is recorded – e.g. length of delay, cause of delay)
     - No

6.4.1 If YES, are they routinely reviewed and addressed to improve unit efficiency?

- - - Yes (please expand)
    - No

1. **Post-procedure**
   1. How much time is allocated for room turnover (time from scope out of patient A to scope into patient B)?
      - 10 minutes
      - 15 minutes
      - 20 minutes
      - Other: _____________
   2. Is turnover time measured?
      - Yes (please explain how – e.g. scope out to scope in, patient out to patient in)
      - No

7.2.1 If YES, what is average turnover time (if available)? Do you have raw data on turnover time that you can share with us?

Average turnover time: _____________

Period of time over which this was measured: _____________

Data available: Yes No

- 1. Please describe the endoscopy room turnover process.
  2. Does the nurse assigned to the endoscopy room receive any help during the turnover process?
     - Yes (please explain how and by whom, e.g. float nurse or porter helps with cleaning)
     - No

1. **Quality improvement**
   1. Have any initiatives been undertaken to decrease room turnover time?
      - Yes (please expand)
      - No
   2. Is any data on performance (e.g. volumes, efficiency, quality measures) shared with unit staff?
      - Yes (please explain what is shared and why)
      - No
   3. Have there been any quality improvement projects to improve efficiency conducted in the endoscopy unit?
      - Yes (please explain what they were trying to improve and how)
      - No
